# On the Treatment of Purpura Hæmorrhagica

**Published:** 1859-09

**Authors:** S. L. Hardy

**Affiliations:** Physician to the Hospital for Diseases of Children, Dublin


*On the Treatment of Purpura Hæmorrhagica. By Dr. S. L. Hardy, Physician to the Hospital for Diseases of Children, Dublin.* (Dublin Hospital Gazette.)

*Idem.*

We notice the observations of Drs. Whitehead and Hardy together, as they refer to the same disease, though presenting under different phases. We quote from Ranking's Abstract the following remarks of Dr. Whitehead:

"A maculated state of the skin is frequently met with in the children of this class of people, consisting of small circular spots, the size of face freckles; not, however, occupying the face and hands as freckles do, but every other part of the body except the feet. They seem to infest parts covered with the clothing, while freckles are only seen on parts exposed. They have a purple color, like the maculæ of typhus, not a pale yellow like freckles, and are unassociated with any form of disease saliently expressed; but they seem, nevertheless, to indicate a depraved habit of body. The skin has a sickly, depraved pallor, the flesh is flabby, the temper fretful, and the energy subdued. The parents appear to regard their presence as natural and unavoidable, as they are never the subject of treatment, nor is attention directed to them when the body is examined for other purposes. They seem peculiar to the lower classes, and are caused by sloth and personal neglect, as they are never met with in the offspring of the cleanly and thrifty. There is no doubt that this macula cachectica is in reality a disease of the skin, due proximately to interrupted cutaneous transpiration. Its cause is uncleanliness. Such children are seldom washed, except the hands and face, oftener than once in many weeks, and even then very imperfectly and without the use of soap. When the children get older, and the washing process is left to themselves, it is still less perfectly done, and the whole body is not washed, in the vast majority of instances, once in several years. In such persons the spots which appeared in infancy are often seen up to adult life."

We would not have transferred so much of this "Report" to the columns of the "Journal," if it were not for the purpose of showing how distorted views and partial statements prove, in medicine, a fruitful source of fallacy. We deny that the disease in question is ever caused by mere uncleanliness, or that "it is peculiar to the lowest classes." Under the title *macula cachectica*, our readers may recognize *purpura simplex*, a disease by no means uncommon, particularly amongst the ill-fed children of the poor. Still, we must not suppose it confined to such. We have seen it in children where neither poverty nor want of cleanliness could be suspected of having any share in its production. Practical men view it as a disease proceeding from a general deterioration of the solids and fluids of the body —one in which there is a change in the crasis of the blood. This is defibrinated, and escapes from vessels that are impaired in their integrity. We do not object to the "washing process" as an important hygienic means, but should think it, by itself, very inadequate, indeed, to remove the cachectic condition of the system. Iron, quinine, and tonics, combined with astringents (having previously administered purgatives), will be likely to prove more efficient remedies, and more consistent with our notions of a rational *methodus medendi*.

In the same number of *Ranking's Abstract*, there are four cases of *purpura hemorrhagica* detailed by Dr. Hardy. These all recovered under the use of tincture of larch bark. The virtues of this tincture are, we presume, due to turpentine and tannin. These certainly have been found useful remedies in such cases, even when administered separately, and there may be some advantages in prescribing them in union, as they are in the tincture. Tannin and gallic acid are, with some, favorite medicines in *purpura*, and more than thirty years ago, turpentine was extolled by Whitlock and Magee (*vide Edinburgh Medical and Surgical Journal*, vol. xviii, p. 540, and vol. xvii, p. 307) for its efficacy in the same disease. The dose of the tincture varies according to age, from eight drops three times daily, to fifteen drops every two hours. It was given in lemonade, which generally constituted the drink of the patient. The food consisted of vegetables and fruit.

